# Supplementary material for: Developing content for a virtual reality scenario that motivates quit attempts in adult smokers: A focus group study with art-based methods
Source: PLOS Digit Health. 2024 May 23;3(5):e0000512. doi: 10.1371/journal.pdig.0000512 (PMC11115299; doi:10.1371/journal.pdig.0000512)
Supplement: S1 Table — (DOCX) [file pdig.0000512.s001.docx]

**S1 Table: REDCap screening survey**

**Baseline Screening Questionnaire for people who smoke.**

| **What is your age (in years)?** | 0-120 |
| --- | --- |
| **Do you smoke cigarettes at all nowadays?** | 1. No 2. Yes |
| **Do you smoke every day?** | 1. No 2. Yes |
| **How many cigarettes per day do you usually smoke?** | 1. I don’t smoke cigarettes every day. 2. 10 or less 3. 11-20 4. 21-30 5. 31 or more |
| **Do you live in London?** | 1. No 2. Yes |
| **Are you willing to attend a focus group session at UCL?** | 1. No 2. Yes |

**Additional baseline questions for eligible participants**

| **What is your name?** | Free text |
| --- | --- |
| **What is your email address?** | Free text |
| **What is your mobile phone number?** | Free text |
| **Which of the following describes how you think about yourself?** | 1. Male 2. Female 3. In another way 4. Prefer not to say |
| **Which of the following best describes your job?** | 1. Manual 2. Non-manual 3. Student 4. Other (e.g. retired, unemployed etc.) |
| **Do you have any post-16 educational qualifications (e.g. T-levels, A-levels, University degree)** | 1. No 2. Yes |
| **What is your ethnic group** | 1. Any Asian or Asian British background 2. Any Black, Black British, Caribbean or African background 3. Any White background 4. Mixed or multiple ethnic groups (e.g. White and Black African or White and Asian) 5. Other ethnic group (e.g. Arab) |
| **Which of the following best describes you?** | 1. I REALLY want to stop smoking and intend to in the next month 2. I REALLY want to stop smoking and intend to in the next 3 months 3. I REALLY want to stop smoking and hope too soon. 4. I want to stop smoking, but I don't know when I will 5. I want to stop smoking but haven't thought about when. 6. I think I should stop smoking but don't really want to. 7. I don't want to stop smoking. |
| **Have you ever made a serious attempt to quit smoking in the past 12 months? By serious we mean you decided that you would try to make sure you never smoked again.** | 1. No 2. Yes, but not in the past year. 3. Yes, in the past year. |
| **Have you ever used any of the following to help you stop smoking? (select all that apply)** | 1. Nicotine replacement product (e.g. patches/ gum/ inhaler) 2. Nicotine replacement product n prescription and given to you by a healthcare professional 3. Zyban (bupropion) 4. Champix 5. E-cigarette or another vaping device 6. Attended a Stop Smoking group 7. Attended one or more Stop Smoking one-to-one counselling/ advice/ support sessions 8. Phoned a smoking helpline 9. A book or pamphlet 10. Visited a website 11. Use an application (app) on a computer, tablet, or smartphone 12. None of these 13. Other. |
| **By a virtual reality (VR) headset we mean an electronic device that you wear on your head with a screen inside that allows you to see seemingly real images in 3D. People commonly use VR headsets to play games, watch films or sport, learn new skills, experience culture, for social media and other activities.**  **Have you ever used a VR headset more than once previously?** | 1. No 2. Yes |
| **What are you preferred dates to come into UCL for a one-off focus group? (select all that apply)** | 1. TBD 2. TBD 3. TBD 4. TBD |
| **Where did you hear about this study?** | 1. Physical poster 2. Facebook 3. Instagram 4. Callforpartiicpants.com 5. Friend/ family recommendation 6. Other |
| **Would you like to receive a plain language summary of the results (for adults who smoke) when ready?** | 1. No 2. Yes |
