# Supplementary material for: Developing content for a virtual reality scenario that motivates quit attempts in adult smokers: A focus group study with art-based methods
Source: PLOS Digit Health. 2024 May 23;3(5):e0000512. doi: 10.1371/journal.pdig.0000512 (PMC11115299; doi:10.1371/journal.pdig.0000512)
Supplement: S2 Table — (DOCX) [file pdig.0000512.s002.docx]

**S2 Table: Question route for focus groups**

| Phase | Questions | |
| --- | --- | --- |
| *Phase 1- Introduction (10 minutes)* | - Introduce the facilitator (TO) and the other researchers in the room. - Explain aims of the focus group with PowerPoint slides: “The goal of the focus group is to get an idea of what content adults who smoke would most like to see in a virtual reality intervention aimed at motivating and encouraging attempts to quit smoking. The results of this study will inform development of a VR prototype”.   - The PowerPoint explains the technical terms such as “content” and “prototype” with examples to illustrate. - Explain presence of the audio recorders. - Go over consent and what this means. Allow participants time to read over the participant information sheet and consent forms again, which they can take home with them. - Go over focus group “ground rules” and confidentiality: “All information collected today will be confidential and no one’s name will be disclosed or linked to any quotes in the final report. As this is a group discussion, please respect the privacy of your fellow participants and not share the contents of this discussion outside of this room. Also, please be respectful of each other’s opinions. I hope this encourages you all to speak openly and thank you all for your participation”. | |
|  | Opening question | - Can we go around the room and introduce ourselves, giving your first name and what interested you to take part in this study? |
| *Phase 2- VR demonstration*  *(30 mins)* | Introductory question | 1. Who here has ever used virtual reality before and what was the experience like? |
|  | *Explain the VR video/app that is going to be demonstrated. Demos will be in two rounds.*  (15mins). Let participants know that if they feel uneasy during the demonstration, they can ask to stop, and the headset will be removed. Participants who are waiting for their turn will be given refreshments.  VR demos:   - ([The Hydrous presents: EXPLORE \| SideQuest (sidequestvr.com)](https://sidequestvr.com/app/10021/the-hydrous-presents-explore). – HTC VIVE and Quest - [MultiBrush on Oculus Quest 2 \| Oculus](https://www.oculus.com/experiences/quest/3438333449611263/) | |
|  | Transition questions | Note: clarify that these questions are about their experience of the technology not necessarily the content of the video/ app shown.   1. What is everyone’s overall impression of the VR experience you have just had? |
|  | Key question(s) | 1. Is there anything you would want to change about the headset and controllers and if so, why? 2. Would a modification to this VR experience make it more interesting and attention grabbing and why? 3. If the cost was not a consideration, would anyone want to use the VR and what would you use it for? 4. How do you feel about the implementation of smoking into VR? Do you think there is potential in it to motivate smokers to quit? 5. How immersive would you prefer this VR scenario to be? Semi or fully immersive? (Fully immersive is the most realistic type of VR and it gives you the perception of existing in another world as if what is happening to you is real) 6. Would anyone want to use VR to help you start an attempt to quit smoking- why or why not? 7. Are there any community settings outside people’s homes that you think smoking or health related VR may be suitable for? (If there are no responses give prompts e.g., GP office) |
| *Phase 3*- *Beliefs about* *quitting smoking*:  *(30 minutes)* | Introductory question | 1. Has anyone ever thought about or considered trying to stop smoking before? |
|  | Transition questions | 1. What was the motivation behind your first quit attempt? 2. Did this lead to a serious quit attempt and for how long? (by serious I mean you decided that you would try to make sure you never smoked again. It might have involved throwing away remaining cigarettes or using products like nicotine gum and patches). |
|  | Key question(s) | 1. How do you feel about giving up smoking? What would be your main concern about quitting this time? 2. Can I ask, if you haven’t attempted quitting, could you give a few reasons as to why you haven’t? 3. How long have you been a smoker for? How do you feel about smoking when you first started compared to now? 4. Would anyone say their motivations have changed over time? And if so, telling us how and why they think it might have? |
|  |  |  |
| Phase 4- *Co-design*  *(30-minutes).* | Introduce final activity. Hand out markers and card paper: “For the final part of the focus group we would like you to consider the points made earlier in the discussion and use them as inspiration to sketch out a virtual reality video. Think about the type of support you would like to receive or questions about quitting you would like answers to. Then, think of a story that would encourage you to quit. Sketch one or two pictures that set the scene. Think about the characters involved and the setting. Here is an example for you [show generic example sketch on PowerPoint slide].  Don’t worry about your artistic ability being judged, the goal of this exercise is to help generate ideas. If anyone isn’t up to drawing, I or one of the other researchers can help you. Alternatively, you can write down your ideas as bullet points on the card. You’ll have around 15 minutes for this activity.” | |
|  | Key questions | 1. Can we go around the room and share our sketches or ideas? Feel free to provide helpful comments on each other’s designs, sharing what you liked or what you would change or add. |
| *Phase 5*- *Debriefing*  *(5 minutes)* | Ending questions | - Does anyone have anything else they would like to share? - Does anyone have any questions? - Clarify any misconceptions (if relevant). - Hand out SSS flyers and give out any remaining refreshments. - Advise participants that they will be emailed their compensation. - Thank them again for participating. |
