# Supplementary figures and images for: Developing content for a virtual reality scenario that motivates quit attempts in adult smokers: A focus group study with art-based methods
Source: PLOS Digit Health. 2024 May 23;3(5):e0000512. doi: 10.1371/journal.pdig.0000512 (PMC11115299; doi:10.1371/journal.pdig.0000512)

**S1 Fig: example of a coded drawing**


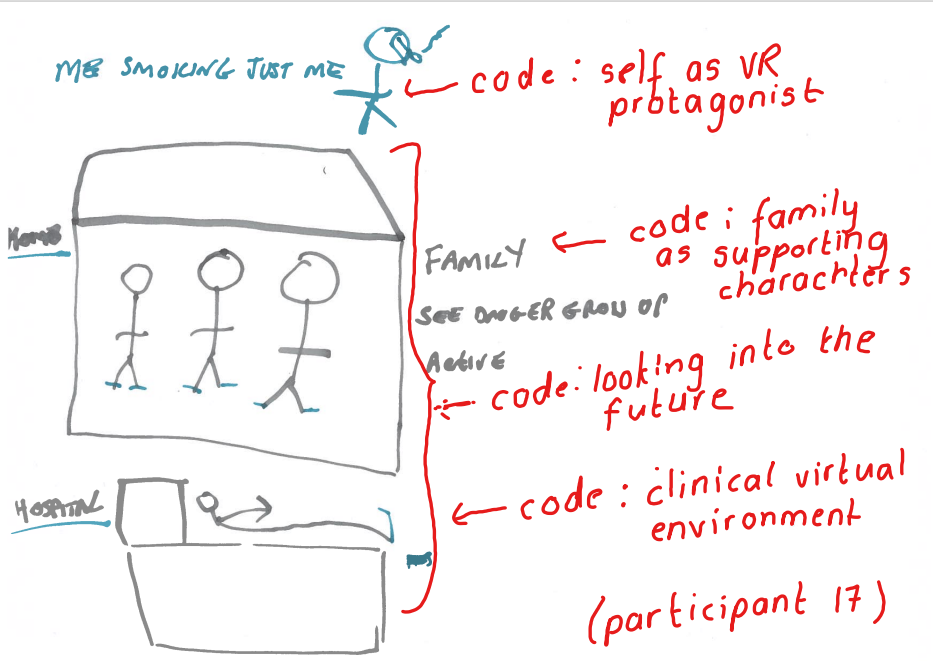

Supplement: S1 Fig — (DOCX) [file pdig.0000512.s003.docx]
