# Supplementary material for: Developing content for a virtual reality scenario that motivates quit attempts in adult smokers: A focus group study with art-based methods
Source: PLOS Digit Health. 2024 May 23;3(5):e0000512. doi: 10.1371/journal.pdig.0000512 (PMC11115299; doi:10.1371/journal.pdig.0000512)
Supplement: Attachment — Submitted filename: Response to Reviewers.docx [file pdig.0000512.s004.docx]

Dear Dr Leo Anthony Celi,

**RE: Response to reviewer comments: PDIG-D-23-00484**

Many thanks to the editor and the reviewers for their helpful comments on our manuscript titled “Developing content for a virtual reality scenario that motivates quit attempts in adult smokers: A focus group study with art-based methods”.

As requested, please find our response to each of the points raised by the reviewers.

| **COMMENTS** | **RESPONSE** |
| --- | --- |
| **Reviewer 1** | |
| In my opinion, the title is quite long, I think it might be interesting to shorten it a bit. | The original title has been retained and includes the study design for completeness. Its length of 139 characters is under the 200-character limit of the journal.  However, in line with the journal format, we have now added a short title, which we had previously omitted: “**Co-design for a smoking cessation virtual reality scenario**” |
| In any case, I wonder if instead of presenting the characteristics of the sample in general in Table 1, it wouldn't be worth having a detailed table with information on each participant. | Given the small sample size and the fact that we collected special category personal data (e.g. race/ ethnicity), we opted not to have a table showing the individual characteristics of each participant. This was to minimise the risk of deductive disclosure.  However, in light of this comment and the subsequent comment on the imbalanced sizes of the focus groups, we have updated Table 1 (add page of updated manuscript that this has been added). The new table has added columns with summary statistics for each of the four groups. We feel this would be beneficial in helping readers see how diverse or homogenous the composition of each group was. |
| the authors explain that they wanted to segment the focus groups by level of motivation, but then they can't do it. I don't see this as a critical point but rather, for example, the imbalance in the number of participants in the focus groups (e.g. group 2 had 10 participants and group 4 had 2 participants) | As mentioned above, we have now updated table 1, to show the breakdown of each individual group.  We added the following to the results section along with the new table **(lines 342 to 343)** (highlighted sections are added revisions):  “The smaller focus groups (groups 1 and 4) were more homogenous than the larger ones in terms of gender, age and educational attainment.”  We have also added a reflection on this point in the strengths and limitations section of the discussion **(lines 731-735)** :  “Due to a combination of oversubscriptions and last-minute cancellations, rather than 6 participants per focus group, the sizes ranged from 2 to 10. We found that the durations of the focus groups were similar (approximately 2 hours), regardless of size indicating that the smaller groups had sufficient interaction. However, for the larger group (n=10), there may not have been enough time for each member to adequately participate and in equal proportion.” |
| In addition, although the paper mentions the inclusion of participants from ethnic minorities and women, it would be beneficial to elaborate on how these demographic factors might have influenced the findings. Are there any notable patterns or differences in perspectives based on ethnicity or gender? | In our analysis, we did not find that responses differed by ethnicity. However, we found that responses differed by age, gender and motivation to quit. Consequently, we have added participants' gender underneath each quote to reflect this better.  In the results we added **(lines 519 to 522):**  Those with VR experience were mostly male. Only two participants owned a headset- participant 1 to play videogames and participant 108 whose sons used it for games too.  In the discussion we added **(lines 674 to 676):**  Most participants with previous VR experience were men, possibly reflecting wider gender patterns of interest in VR. Previous UK survey data indicated that a higher proportion of men compared to women were interested in experiencing VR (55% vs 40%) |
| I missed having, in a **very concrete and pragmatic way, some guidelines and requirements** for the development of this VR intervention based on these findings. | In the discussion under “Implication for future research and practice we have added the following”, which was hinted at but not collated together **(lines 737 to 747).**  **Design requirements:**  Based on the results of this study we suggest that people developing VR interventions for smoking cessation consider including the following:   - Content emphasising the future health consequences of smoking or the benefits of cessation. - The use of graphic imagery to evoke emotion when highlighting the negative impact of smoking. - Emotive storytelling through techniques such as including important others (e.g., family members) as supporting characters in the narrative. - Options that allow the user to interact with and be immersed in the virtual environment, such as prompts to make you move around in the virtual environment and sound effects - Content that can be tailored to meet the needs of the specific user. - Follow-up cessation support after the VR content is viewed. |
| **Reviewer 2** | |
| The issues related to socioeconomic disparity, potential digital exclusion bias, and the homogeneity in focus groups should be carefully addressed. | In the discussion, we have now highlighted how the majority of the sample had post-16 educational qualifications and the implications for socioeconomic disparity **(lines 716 to 721):**  “We were unable to reach the goal of having half the sample from a less advantaged SEP- while occupations were varied, the majority of participants had higher educational attainment (post-16 qualifications)…This may impact the generalisability of findings to all subgroups, especially those who are most socially disadvantaged.” |
| Additionally, providing more clarity on certain aspects, such as the potential long-term efficacy of VR interventions, could strengthen your manuscript. Consider conducting **additional statistical analyses** and providing more context where necessary. This will help ensure the credibility and generalizability of your findings. | We have added a note in the discussion that future work will be needed to assess the long-term efficacy of VR interventions for smoking cessations (e.g. RCTs). However, in our introduction, we did highlight results from smaller pilot studies.  We did not perform any inferential statistical analyses on our data as the main purpose of the screening survey was to describe our sample. Also, the small sample sizes common in qualitative research is not compatible with the larger samples recommended for adequately powered quantitative research. |
| Demographic Representation: While there is diversity in gender and ethnicity, other demographic factors such as socioeconomic status and educational background are not explicitly mentioned. These factors could impact the generalizability of the findings. | This has now been elaborated upon in the discussion **(Lines 708 to 722):**   - mostly balanced according to gender, occupational status, VR experience, and previous use of a digital smoking cessation aid. It was also ethnically diverse. While there was a slightly higher proportion of participants with a low motivation to quit, this mirrors current patterns in England, where the majority of smokers are not highly motivated to quit (2). - - while occupations were varied, the majority of participants had higher educational attainment (post-16 qualifications). - This may impact the generalisability of findings to all subgroups |
| Focus on Fear Appeals: While fear appeals were suggested as a component, relying too heavily on anxiety-inducing content may raise ethical concerns and could potentially lead to unintended negative consequences. | Thank you. We have discussed the pros and cons of fear appeals in the discussion, for example, how the literature suggests how fear appeals are best when paired with content that boosts self-efficacy or how fear appeals can lead to defensiveness. |
| Need for Expert Input: While participants generated ideas, the study acknowledges the need for future research to refine these ideas with experts in VR design and smoking cessation. This raises questions about the feasibility and effectiveness of the proposed VR content. | While the next stage of the project plans on getting input from experts, we feel that the results of this study stand on their own. In line with MRC guidelines and the person-based approach, an important part of intervention design is collecting the viewpoints of the target audience as “experts of their own experience.” This is a perspective distinct from industry professionals. |
| Motivation to Quit: The study mentions that more than half of the participants had low motivation to quit (61.0%). This might impact the generalizability of findings to individuals with higher motivation to quit smoking. | As mentioned previously regarding an earlier comment, cross-sectional data from England suggests that the majority of smokers are not highly motivated to quit. Therefore, the results of this study would be relevant to a large proportion of the English smoking population, though may not be relevant to individuals with higher motivation to quit. We have added a note on this in the discussion **(lines 711 to 714):**  While there was a slightly higher proportion of participants with a low motivation to quit, this mirrors current patterns in England, where the majority of smokers are not highly motivated to quit (2). However, the results of this study may not be generalisable to very motivated smokers. |
| Limited Justification for VR Choice: While the benefits of VR are highlighted, there is a lack of explicit justification for choosing VR over other digital health interventions. A brief comparison could strengthen the rationale.  Assumption of Greater Emotional Response in VR: The claim that VR can produce stronger cognitive and emotional responses than traditional media lacks direct evidence within the context of smoking cessation interventions. | While cited, we did not include the results of that citation in our justification for choosing. We have now done so and added a further study to strengthen our argument **(lines 107 to 110):**  For example, one study demonstrated that VR induced stronger emotional experiences (excitement, nervousness, hostility, and jitteriness) and physiological arousal in users compared to 2D video (11). In the context of smoking, previous VR interventions have resulted in a greater increase in motivation to quit, when compared to cigarette package warnings and 2D videos (6). |
| Digital Inequalities Generalization: While the study acknowledges digital inequalities, it generalizes the risk without specific evidence or data related to the target population's access to VR technology. | In our introduction, we have now added statistics from British survey data on ownership of headsets and interest in VR **(lines 115 to 117).**  Only 4% of British households own a VR headset and previous survey data suggests that up to 37% of individuals have little interest in VR (14,15). |
| Complex Terminology: The introduction contains complex terminology related to behavior models (COM-B, PRIME) and theories, potentially making it less accessible to readers without a background in these concepts. | We appreciate that due to its theoretical underpinnings, this study includes some complex terminology. We have modified the introduction to explain these terms more simply to aid readability (**lines 97 to 100**).  According to PRIME theory, plans formed to quit smoking are based on evaluations (reflective thoughts on what is good versus bad, beneficial versus harmful etc.) of smoking. Evaluations influence, and are influenced by, motives (wants or needs) to continue smoking or quit. |
| Lack of Preliminary Findings: The introduction doesn't provide any preliminary findings from the pilot studies or initial data collection, leaving the reader curious about the initial insights that prompted the current research. | While we originally cited previous VR studies for smoking cessation, for added clarity we have now included some of the main results in our introduction **(lines 87 to 90)** :  “In a previous pilot study testing VR for encouraging quit attempts among unmotivated smokers, the VR group had a greater increase in willingness to quit compared to the two comparator groups (differences in increase of 3.05 and 2.06 on the contemplation ladder of smoking cessation that ranges from 0 to 10, p<0.0001) (5). In another pre/post study, 23 motivated and unmotivated smokers were shown one of two VR scenarios. At one-month follow-up 14 had made a quit attempt (6).” |
| Minimal Discussion on Ethical Considerations: There is limited discussion about potential ethical considerations associated with using VR, especially when incorporating fear appeals or anxiety-inducing content. | In our discussion we have added the following section on participants’ experience of VR **(lines 671 to 673):**  However, a potential downside of VR is its ability to induce motion sickness or cybersickness in users. This could lead to uncomfortable viewer experiences, especially if graphic content were to be used (58). In this study, no participants spontaneously reported any adverse events during the VR demonstration. |
| Research questions:  Limited Scope: The research questions are relatively narrow and might benefit from expansion to include a broader range of factors influencing smoking behavior or perceptions of VR interventions.  Potential Bias in Framing: The framing of the questions assumes a positive stance toward VR content, potentially introducing bias. A more neutral or exploratory framing could encourage a wider range of responses.  Assumption of Homogeneity: The questions imply a degree of homogeneity within the adult smoker population, assuming that their beliefs and views can be addressed collectively. Acknowledging potential diversity within the group may provide a more nuanced understanding.  Missing Timeframe Reference: The questions lack a reference to a specific timeframe, which could impact participants' responses. For example, attitudes toward VR might evolve over time, and specifying a timeframe could capture dynamic changes. | In response to these suggestions, we have modified our research questions for clarity to address the missing time frame, assumption of homogeneity and bias in framing. Also, as mentioned in our introduction, the term acceptability as we have used it (cited from the literature) incorporates both negative and positive aspects **(lines 155 to 158).**   1. As of 2023, what VR content, if any, would address the beliefs of adult smokers and be acceptable to them? 2. In 2023, what were the various views of adult smokers on VR generally and using VR to encourage a quit attempt?   We are reluctant to change the research questions fundamentally as the data has been collected under these questions, which were pre-registered. |
| Limited Exploration of Barriers: The questions primarily explore positive aspects (acceptable content, views on VR), but there is a lack of explicit exploration of potential barriers or challenges that smokers might foresee in adopting VR interventions for smoking cessation.  Potential for Social Desirability Bias: Participants might be inclined to provide responses that they perceive as socially desirable, especially if the questions assume the benefits of VR for smoking cessation. Strategies to mitigate social desirability bias should be considered. | The last theme is based on the main barrier identified by participants, which was that the VR intervention, if intended to be viewed once, would be a “temporary fix” and would not help them sustain a quit attempt long-term.  We included in Table 2, some codes on barriers, but for readability did not expand on them in detail in the main results section. We have now elaborated, particularly on the barriers to using VR at home and participants' ease of use with VR **(lines 532 to 541).**  Given that all participants, except two, did not own a VR headset, there were concerns about the widescale availability of such an intervention. The main barriers identified for using VR at home were the cost of headsets and differential ease of use between users. During the VR demonstration, some participants found the headset and controllers easy to use, while others initially struggled.  *“Yeah, it was good fun and easy to use”.* **-P17 35-44, low motivation, man.**  *“I don’t know if it’s just that I’m bad at gaming controls, that I found it a bit confusing at first. I mean, I think once you get the hang of it, it’s really fun”.* **- P136 18-24, low motivation, woman.**  *“I like it, even though…. I didn’t get how to use it straight away”* **-P92 18-24, low motivation, woman.**  Some participants suggested that a smoking VR scenario should first have a tutorial page to familiarise less experienced users with the controllers.  During data collection, we encouraged participants to give their true opinions, regardless of whether they were positive or negative. We have now clarified this in our reflexivity section **(lines 317 to 320):**  “However, during the focus groups, T.O. tried to de-emphasise the project's overall goal (developing a VR smoking cessation intervention) and emphasised that both negative and positive answers were welcome. This was to prevent participants from feeling obligated to give favourable answers (social desirability bias)”. |
| Sequence of Questions: The sequence of the questions could influence responses. For example, asking about acceptable content before exploring general views might shape participants' perceptions. | The order of the research questions does not reflect the order of questions in the focus group. The full question route for the focus group is in the supplementary material and an outline is given the methods. We started with exploring general views on VR and smoking cessation separately before moving on to asking about content and the co-design activity. |
| Potential Group Censorship: The acknowledgment of the potential for participants to censor themselves in a group setting, particularly on sensitive topics, raises concerns about the completeness and authenticity of responses. | For all themes, we were able to give both the majority and minority opinions for example:   - Positive vs negative smoking identity - Wanting to quit vs no plans to quit - Quitting because of family vs being internally motivated - Finding the VR easy to use vs difficult to use - Positive scenarios vs negative scenarios   These contrasting opinions were found between groups and within groups, suggesting that participants had a level of comfort to (politely) disagree with each other or give an opposite opinion. However, there is always the possibility of some censorship in any kind of group discussion, hence our reflection in the methods **(lines 171 to 173)** . |
| Artistic Abilities Concerns: Participants' potential nervousness about their artistic abilities being judged might introduce bias in the data, impacting the free expression of ideas through art-based methods. | Participants were given the option to write down bullet points instead of drawing. Also, participants explained their ideas verbally after the drawing session. Therefore, any rough/ less detailed sketches could be elaborated on during the group discussion and this was part of the transcript.  **(Lines 261 to 262):**  The facilitator provided participants with markers and paper and asked them to use the discussion points raised in phases 2 and 3 to draw ideas for VR still frames they thought would increase their motivation to quit smoking. Participants could write bullet points if unable to draw. Participants shared their designs verbally with the group. |
| Small Sample Size: While the study aims for theoretical saturation, the small sample size (N=24) might limit the generalizability of findings, and achieving true saturation may be challenging. | We have now included this limitation in the discussion, **(lines 715 to 717):**    “While we recruited fewer participants than originally planned, when analysing the last focus group, no new themes were developed. However, a larger sample size would have ensured true theoretical saturation.” |
| Assumption of VR Familiarity: The VR demonstration assumes a basic familiarity with VR technology, which might influence participants' responses and could be a potential bias if some participants are not familiar with it. | Before the focus groups, the screening survey asked each participant whether they had used VR more than once. As recorded in table 1, this was 42.9% of participants.  We have clarified the data collection section of the methods as follows:  **(lines 250 to 254 )**  *Phase 2-VR demonstration*: As some participants may not have used VR before, we wanted to briefly familiarise them with the technology.  In the question guide (supplementary material) …… Participants were asked about their reactions to the VR demonstration, including what they did and did not like and if it was easy or difficult to use.  As mentioned earlier we have also included some quotes from the codebook, on how easy or difficult the VR demonstration was. |
| Limited Exploration of Negative Aspects: The focus on positive aspects of VR content creation might overlook potential negative perceptions or barriers participants could have encountered. | In the supplementary material (question route) we did ask participants about (after the VR demo):   - Overall impressions of the VR - Any modifications they would like to make? - If cost was not a consideration, would you use VR again? Why or why not?   These questions are neutral and we invited participants to talk about the negatives as well as the positives. |
| Changes to Pre-Registration: The deviation from the pre-registered plan regarding segmentation of focus groups based on motivation to quit raises concerns about potential biases introduced by this change. | Ideally, we would have preferred to segment groups by motivation to quit. However, practically it was difficult to find days/ and times with enough participants available. From an ethical perspective, considering that a large number of participants had already invested time into the recruitment process (i.e. reading and filling out multiple forms) we wanted to honour that commitment and invite them to the focus groups, whilst making the best use of resources. |
| Positionality of Researcher: The researcher's identity as a non-smoker and potential "outsider" status might influence the dynamics within the group, introducing a level of social complexity. | We have acknowledged in our reflexivity section how the characteristics of the moderator can influence the data generation process. Multiple identities can be held at the same time (e.g., age, racial identity, smoker identity). Given we wanted a diverse sample, it would be impossible to have a moderator who matches every participant on every characteristic. However, while being a “non-smoker” is of particular relevance to this study, only participants who directly asked were aware of this. We have clarified this in the manuscript. (**Lines 313 to 314):**  However, some participants (who directly asked) knew that T.O. was a non-smoker. |
| Limited Exploration of Motivation Over Time: The study mentions the dynamic nature of behaviour and motivation but does not explicitly address potential changes in participants' motivation over time, which could be crucial for understanding the effectiveness of interventions. | During data collection, we specifically asked participants how their motivation to quit and smoking behaviour has changed over time. For example (full question route is available in the supplementary material):   - How long have you been a smoker for? How do you feel about smoking when you first started compared to now? - Would anyone say their motivations have changed over time? And if so, telling us how and why they think it might have?   While the factors influencing motivation over time were in our codebook, we did not initially report our themes to include this nuance in the main manuscript. We have now done so **(for example lines 444 to 455) :**  Furthermore, in line with “coming of age” stories, in much of the suggested content, family members were recommended as supporting characters (figure 3, sketches, B, C and D). This likely reflects how for the majority of participants, their motivation to quit had changed over time, due to changing family circumstances. For example, participants who had become parents expressed that their motivation to quit had increased because they wanted to live longer for their children and grandchildren.  *“Yeah, for me, the main difference is that I’m a family man now and obviously with a daughter, it's centred around her.”* **-P17,** **35-44, low motivation, man.**  Seeing younger family members start smoking was also a factor that changed a small number of participants' views towards smoking.  *“I have, little brothers and they both started to smoke… It's quite shocking when you have somebody who you care about smoking and your different attitude towards that… I see them smoking now. And I'm like, “Why did I do that?””-* **P165, 35-44, high motivation, woman.**  . |
| Focus on Qualitative Analysis: The emphasis on reflexive thematic analysis and critical realism suggests a predominant focus on qualitative data, potentially relegating the quantitative data to a supportive role. There is a need for a more explicit integration of both types of data. | As part of the larger project, we have included quantitative analysis in the form of cross-sectional population studies. However, the focus of this study was qualitative. |
| Single Measure for Motivation: The use of the MTSS provides a single-dimensional measure of motivation. Incorporating additional measures or exploring motivation from different perspectives could enhance the understanding of participants' readiness to quit. | We acknowledge that there are other scales to measure motivation to quit, though we used a validated scale of motivation to quit. Research comparing Stages of Change, the Motivation To Stop Scale, and a Likert scale showed comparable predictive and construct validity as measures for intention to quit smoking (Hummel K, Candel MJJM, Nagelhout GE, Brown J, van den Putte B, Kotz D, Willemsen MC, Fong GT, West R, de Vries H. Construct and Predictive Validity of Three Measures of Intention to Quit Smoking: Findings From the International Tobacco Control (ITC) Netherlands Survey. Nicotine Tob Res. 2018 Aug 14;20(9):1101-1108. doi: 10.1093/ntr/ntx092. PMID: 28472427; PMCID: PMC6093485.) and has been shown to be highly predictive of subsequent quit attempts among the smoking population in England (https://pubmed.ncbi.nlm.nih.gov/22943961/) |
| Limited Discussion on Validation: While the MTSS is mentioned as a validated measure, there is limited discussion on the validation process or the psychometric properties of the measure within the context of this study. | Under measures we have now added the following **(lines 218 to 226):**  In a previous study to assess the MTSS’ predictive validity among the English smoking population, the accuracy to discriminate between smokers who did and did not attempt to quit had an area under the curve (ROC_AUC_) of 0.67 (95% CI = 0.65–0.70), which is considered to be broadly acceptable (36). Research comparing Stages of Change, the MTSS, and a Likert scale showed comparable predictive and construct validity as measures for intention to quit smoking (38). |
| Inconsistency in Sample Size Reporting: There is an inconsistency in reporting the sample size, with the text indicating a final sample of 21 participants, while the table suggests 26 participants. Clarification and consistency are needed. | Table 1 has been modified in response to reviewer 1’s suggestions. The new table should not have any typographical errors. |
| Limited Exploration of Quantitative Data: The results primarily focus on qualitative findings, with limited exploration of quantitative data from Table 1. A more in-depth analysis of quantitative results, including patterns and trends, would provide a more holistic view. | As mentioned earlier: We did not perform any inferential statistical analyses on our data as the main purpose of the quantitative data collected in this study was to describe the sample. Also, the small sample sizes common in qualitative research is not compatible with the larger samples recommended for quantitative research to produce statistically significant results.  However, as part of the study's larger project, we have a quantitative population-level study to examine interest in VR, which will be published separately. Future work will collate all these sources of evidence. However, we feel this study stands on its own as user involvement is an integral part of the person-based approach to intervention development. |
| Discussion of Participant Skepticism: While there is mention of some participants being skeptical about VR interventions, a more in-depth exploration of potential barriers, concerns, or reservations would enhance the discussion. | We have expanded the theme “VR as a health service” to elaborate on the code mentioned in Table 2 “barriers to using VR at home” **(lines 532 to 541).**  Given that all participants, except two, did not own a VR headset, there were concerns about the widescale availability of such an intervention. The main barriers identified for using VR at home were the cost of headsets and differential ease of use. During the VR demonstration, some participants found the headset and controllers easy to use, while others initially struggled. |
| Further Elaboration on Themes: While the themes are well-presented, further elaboration on the implications of each theme and their potential impact on designing effective VR interventions would strengthen the discussion. | We have now included a new section in the discussion under “implications for future research and practice”, with concrete suggestions on how the themes will influence the development of the intervention moving forward **(lines 740 to 750).** |

Kind regards,

Tosan Okpako
