# Supplementary material for: Developing content for a virtual reality scenario that motivates quit attempts in adult smokers: A focus group study with art-based methods
Source: PLOS Digit Health. 2024 May 23;3(5):e0000512. doi: 10.1371/journal.pdig.0000512 (PMC11115299; doi:10.1371/journal.pdig.0000512)
Supplement: Attachment — Submitted filename: Response to Reviewers round 2.docx [file pdig.0000512.s005.docx]

Dear Dr Leo Anthony Celi,and Dr Haleh Ayatollahi

**RE: Response to reviewer comments: PDIG-D-23-00484**

Thanks to the editor and the reviewers for their comments on our manuscript titled “Developing content for a virtual reality scenario that motivates quit attempts in adult smokers: A focus group study with art-based methods”.

The previous reviewers appeared satisfied with the responses to their comments. As requested, please find our response to each of the points raised by the second round of new reviews.

| **COMMENTS** | **RESPONSE** |
| --- | --- |
| **Reviewer 4** | |
| There is no rationale for the chosen sample size | We based our sample size on theoretical saturation. However, we have added some extra explanation (new parts highlighted in yellow):  **Line 226-228:** “This study aimed to recruit six participants for four different focus groups (N=24), to achieve theoretical saturation, (i.e. when collection and analysis of additional data does not provide new insights or themes.) |
| There is a skew toward younger, digitally connected individuals | We have added a few lines to the limitation section.  **Line 777-781:** “The sample was skewed towards younger, digitally connected participants. Also, as the study was predominantly advertised through online methods, there is a possibility that this study does not include the viewpoints of those who are most digitally excluded and without regular internet access. This may impact the generalisability of findings to all subgroups, especially those who are older or most socially disadvantaged.” |
| Discuss how the researcher’s factors were managed during the focus groups and the reflexivity strategies employed during data analysis.  Provide information on focus group moderation techniques and strategies to mitigate bias. | Thank you for this suggestion. Some strategies were already provided. However, we outlined additional moderation strategies.  **Line 353-360:** “However, during the focus groups, T.O. tried to de-emphasise the project’s overall goal (developing a VR smoking cessation intervention) and emphasised that both negative and positive answers were welcome. This was to prevent participants from feeling obligated to give favourable answers (social desirability bias). Occasionally, the moderator gently asked quieter participants if they would like to contribute, but otherwise left the conversations to proceed organically. Participants were also given ground rules before the discussion, regarding respecting alternative viewpoints and listening to others. They were also reassured that if they became distressed, they could leave the conversation at any time and still receive compensation for their time. No participants left the focus group prematurely and no adverse events were reported.”  Also we added:  **Line 331-332:** “To minimise bias in the interpretation of results, the wider research team, made suggestions as to which codes needed more evidence from quotes in the transcripts.” |
| Elaborate on potential limitations arising from pre-registered protocol modifications and how these changes affect the study's validity and generalizability. | The only modification from the protocols were sample size distribution, and segmenting groups by motivation to quit. We touch on this in the discussion.  **Line 782 -790:** “Additionally, we were unable to segment the focus groups by motivation to quit. Some authors suggest that more homogenous focus groups increase participants’ ability to speak freely (27). This would increase the credibility of the data produced (i.e., the extent to which findings represented the true ideas of the participants) (49). As smoking can often be stigmatised due to its negative health effects, we initially posited that smokers with lower motivation to quit may feel inhibited if in a room with high-motivation smokers expressing anti-tobacco views (68). For example, in this study, only one participant expressed an explicitly positive smoker identity. It is possible that other participants felt unable to do so. However, the majority of participants were open about their struggles to quit or their plans to continue smoking, which suggests that there was some level of comfort.”  Also:  **Line 792-796:** “Due to a combination of oversubscriptions and last-minute cancellations, rather than 6 participants per focus group, the sizes ranged from 2 to 10. We found that the durations of the focus groups were similar (approximately 1.5 to 2 hours), regardless of size, indicating that the smaller groups had sufficient interaction. However, for the larger group (n=10), there may not have been enough time for each member to adequately participate and in equal proportion.” |
| Describe the recruitment process. | We describe the recruitment process here:  **Line 206-209:** “Participants were recruited through digital posters advertised on Facebook and Instagram, Callforparticipants.com and physical posters displayed around University College London’s (UCL) campus. The poster included a link to an online screening survey (Supporting information table 1), which was administered via the REDCap electronic data system (embedded within UCL’s Data Safe Haven) (35).”  **Line 221-223:** “All eligible participants, who completed the survey, were contacted by email to schedule a group slot. They were also contacted by phone a day before the focus group to confirm their attendance.”  We also provide a flow diagram of the recruitment process (see Figure 1). |
| Discuss limitations related to the use of VR technology, such as accessibility issues and the need for specialized equipment. | **Line 728-731:** “As part of this study’s larger project, a future study with smoking cessation professionals will explore their opinions on the possible barriers and facilitators of incorporating VR into a healthcare setting, including accessibility issues and the need for specialised equipment.” |
| Discuss safeguards to protect vulnerable participants and ensure equitable access to VR interventions. Ethical oversight should extend beyond the study period to address potential long-term effects on participants' well-being and autonomy. Were there follow-up assessments and ethical monitoring post-intervention? | Strategies to improve access to future VR interventions are discussed in the paper, in particular under the theme titled “Smoking cessation VR as a health service treatment.” The main suggestion was to have VR in community health settings to circumvent the lack of home ownership.  This study was deemed low risk by the UCL ethics committee. In line with recommendations from the ethics committee, we provided participants with smoking cessation leaflets after the focus group, which were optional to take home.  **Line 367-371:** “Ethical approval was granted by UCL’s Research Ethics Committee (ID: 25627.002). Participants provided informed consent. Identifying information, such as participants’ names, was removed and data was stored securely. Participants had the option to collect smoking cessation leaflets and business cards after each focus group. Participants were given the contact details of the UCL ethics committee to report any concerns. No concerns or complaints were reported after the focus groups.”  There were no follow-up assessments or ethical monitoring post-intervention. This study was minimally invasive, and the VR games demonstrated were similar to games they would encounter in a non-research setting. Also, participants only consented to attend the focus group, they did not provide permission to be contacted further, apart from being emailed a summary of results. Therefore, further contact could be perceived as intrusive at this stage. |
| Discuss potential synergies between VR interventions and pharmacotherapy. | Participants discussed the type of advice they would like to receive, regarding nicotine replacement therapy. However, the intervention that this study is co-designing for is a VR video, intended to be viewed once by participants, to encourage them to make an attempt at quitting.  Discussing synergies between VR and pharmacotherapy may be more suited to interventions such as VR-cue exposure therapy and is beyond the scope of this paper, given the current length of the manuscript. |
| Discuss the safety concerns of integrating VR interventions into smoking cessation treatment plans and how it would help pharmacists. | Safety concerns are discussed in the discussion:  **Line 715-718:** “A potential downside of VR is its ability to induce motion sickness or cybersickness in users. This could lead to uncomfortable viewer experiences, especially if graphic content were to be used (60). In this study, no participants spontaneously reported any adverse events during the VR demonstration. However, the views of experts in VR design will be sought to balance any potential harms with maximising engagement”.  The views of health professionals such as pharmacists are being sought in a separate study.  **Line 728-731:** “As part of this study’s larger project, a future study with smoking cessation professionals (such as doctors, stop-smoking advisors and pharmacists) will explore their opinions on the possible barriers and facilitators of incorporating VR into a healthcare setting, including accessibility issues and the need for specialised equipment.” |
| Discuss potential counterarguments and alternative perspectives on the ethical and existential dimensions of VR-based smoking cessation interventions. | We felt that this reflection was most relevant to content suggested by participants that focused on pleasurable and brightly coloured images to reduce stress linked to smoking. The following has been added to the discussion.  **Line 743-754:** “Some participants suggested using VR for long-term cessation support. The content drawn involved pleasurable images that were “exotic,” “euphoric” and brightly coloured. The aim was to reduce anxiety and stress, which were common triggers for smoking. However, this type of content may also bring its own set of ethical concerns. Numerous scholars and philosophers are concerned with the psychological and existential dimensions of VR (70). Some authors argue that prolonged exposure to immersive technologies and virtual worlds could lead to users feeling dissatisfied with the real world and potentially developing depressive symptoms (71). This would be counter-productive to the intentions of the participants who suggested this type of content. A study conducted in 2009 found that exposure to VR increased dissociative experiences in participants and lessened their sense of presence in actual reality (71). However, a description of the VR content shown was not included in that study. While outside the remit of this project, future work could explore the usefulness and implications of content based on pleasurable images in treating substance use, (including and beyond smoking) and mental health more generally”. |
| **Reviewer 5** | |
| Authors have proposed an interesting topic, but the methods chosen to treat it are neither rigorous nor appropriate. The results obtained are not significant, as they derive from the comparison of non-homogeneous groups in terms of number, age, daily cigarette consumption and familiarity with VR environments, all elements that characterise and heavily influence the results. Authors are encouraged to extend their research and show robust and rigorous results.  Furthermore, the different desire to quit smoking in the participating subjects, the support received from the family context, and the custom motivational elements present in the VR environment are all factors that influence in a different, but not independent way, the level of patients' engagement with respect to the chosen method. This prevents the motivational results from being delegated solely to the VR environment chosen for each patient. Authors are invited to standardise the chosen approach, so that there can be no doubt about the causes that determine the results shown. | Thank you for this comment. However, this is not a causal study, such as a randomised control trial or before and after comparison. Participants did not receive a smoking cessation VR intervention (e.g., they did not receive VR-based family support, rather they suggested through sketches that it may be useful). The VR intervention has not yet been developed.  Rather, the goal of this study was to collect ideas from current adult smokers about the type of content they would like to see in such an intervention.  As noted in the introduction, when designing new interventions, it is recommended that the views of the target population are included. We have attached the section of the introduction detailing this below. Qualitative work (such as focus groups or interviews) is recommended as they provide more in-depth data than other methods of data collection such as surveys.  **Line 150-155:** “The role of acceptability within public health is reflected in the Medical Research Council’s (MRC) guidance for the development and evaluation of complex health interventions and the person-based approach (PBA) (19,20). The MRC guidance recommends formative development work to understand the views, preferences, and behaviour of the target population (19). Using the PBA provides a framework to ground the development of a VR intervention in the perspectives of the people who will eventually use it, thereby maximising its acceptability and potential efficacy (20). “  Also, as this is a qualitative (not quantitative) study, statistical concepts such as “significance” do not apply. In qualitative research that uses thematic analysis, the robustness of the themes comes from rich description and the use of quotes as evidence. We purposefully designed the study to have a non-homogeneous sample (i.e. diverse sample) to get a range of different viewpoints during the focus groups. In our themes, we detail how viewpoints differed based on factors such as age and familiarity with VR. |
| **Reviewer 6** | |
| Give the abstract a dry and clear writing that the rest of the manuscript also has. | We have made some minor changes for extra clarity and shortened some sections. We would be willing to act further on any specific suggestions for additional changes.  **Line 24-44:** “Virtual reality (VR) could be used to deliver messages to smokers that encourages them to attempt quitting. For a VR smoking cessation intervention to be effective, the target population must find the content engaging, relevant, inoffensive, and compelling. Informed by health behaviour theory and narrative transportation theory, this study used focus groups combined with art-based methods (participant sketches) to inform the development of VR content that will appropriately address smokers’ beliefs about quitting smoking. Data were analysed using reflexive thematic analysis. Four in-person focus groups (N=21) were held between July and August 2023. Just under half the sample were from an ethnic minority (42.8%) and women (42.9%), and the mean age was 33.6 years (standard deviation = 15.9). More than half the sample had a low motivation to quit (61.0%). We developed six themes concerning: the VR content suggested by participants, the rationale behind it, its technological execution and potential widescale implementation. Many participants downplayed the health consequences of smoking, prioritising the immediate rewards of smoking over quitting’s long-term benefits. Therefore, participants suggested content set in the future, showing the benefits of cessation or the negative consequences of continued smoking. Family members were recommended as supporting VR characters to increase the content’s emotional salience. Participants also suggested graphic content that would trigger anxiety about smoking, suggesting that fear appeals were welcome. Participants wanted a truly novel intervention- not a leaflet about smoking statistics presented through VR. Participants suggested healthcare locations (e.g., doctors' offices) for implementation, as home ownership of VR headsets is low. Also, this would make the VR appear more legitimate as a health intervention (rather than casual entertainment) and could complement in-person advice. Future research will refine the participant-generated ideas with experts in VR design and smoking cessation.” |
| The works of Michie et al. are very interesting in the field of theories that explain change, addiction, and behavior, but it could also be interesting for the reader and strengthen the manuscript to know the theoretical references prior to the team's work, and I refer in particular to: the biopsychosocial model and theories of Health Psychology, Theory of Planned Behavior, Social Cognition, Transtheoretical Model, Social Reinforcement Model, and Self-Efficacy Model. These models are just an example that could make the introduction more complete and organic compared to the excellent work done later. | Thank you for this comment. We placed a particular emphasis on COM-B and PRIME as these were the theories that informed the deductive parts of the thematic analysis. As this paper is already quite long (almost 10,00 words) we have included a brief overview paragraph to the introduction and other additions.  **Line 96-103:** “Many prominent theories and models exist which seek to explain smoking as a behaviour and the requirements needed for that behaviour to change (i.e. making a quit attempt). The determinants of smoking are a mixture of psychological, biological, and sociocultural factors (7). Theories of smoking cessation differ in their conceptualisations of the relevant importance of these factors and how they are interlinked. Examples of prominent theories include but are not limited to, “The Health Belief Model”, “Social Cognitive Theory” “The Theory of Planned Behaviour” and “The Transtheoretical Model (Stages of Change)” (7–9). However, this study uses COM-B and PRIME theory…”  **Line 217-220:** “PRIME theory promotes offering cessation support to smokers, regardless of their current level of motivation to quit (13). This is in contrast to the transtheoretical model, which posits that smokers with different readiness to change (stages) require different interventions. For example, only those who are most ready to quit should receive advice on cessation medication (8,40).” |
| R251-252: A non-expert reader might find it interesting to read an explanation of the environments on which these VR games are based or a small note on the sources that report basic information released by the manufacturing company | The links to the VR demonstration games were originally in the supplementary material (the topic guide). They have now been added to the main manuscript.  **Line 271 -277:** *“Phase 2-VR demonstration*: As some participants may not have used VR before, we wanted to briefly familiarise them with the technology. Participants played one of two VR games. The first was a 3D painting game in an abstract virtual environment called “Multibrush” on PICO 4 Enterprise (MultiBrush on Meta Quest \| Quest VR games \| Meta Store). The PICO 4 Enterprise headset retails at approximately £900 ([PICO 4 Enterprise EU (8G+256G) VR Headset (smartteck.co.uk)](about:blank). The second was an interactive video about marine wildlife called “Hydrous presents: EXPLORE” on HTC Vive ([The Hydrous presents: EXPLORE \| SideQuest (sidequestvr.com)](about:blank).” |
| R363-364: At this point, it could be interesting to make a reference to the transtheoretical model to explain the importance of maintenance and avoidance of relapse. | We have added to the discussion section. We do not go into great detail because we believe there are various reasons that the TTM is not particularly applicable to smoking cessation (E.g., see <https://onlinelibrary.wiley.com/doi/10.1111/j.1360-0443.2005.01139.x> ; and one of us co-authored a book, which elaborates this point further in Chapter 9 – West and Brown, Theory of Addiction). The above-mentioned paper is also included in the reference list of this manuscript.  **Line 736-738:** “They saw the VR intervention as a temporary fix. The transtheoretical model of smoking cessation highlights the importance of behavioural support and NRT during the maintenance stage to prevent relapse (8).” |
| R419: It does not seem that the link clearly and easily contains the package of sketches of the participants. | Thank you for pointing this out. The link should now contain this. |
| R519-520: An expert reader might find it interesting to know how many minutes the demonstration lasted. Generally, an inexperienced gamer, on the first use, has difficulty using the headset for more than 20/40 minutes consecutively. Was the demonstration in this range or was it lower or higher? | **Line 283- 284:** “Each participant chose which demonstration they would prefer and spent approximately 5-7 minutes playing their chosen game.” |
| In a general reading, a less experienced reader might be interested in understanding the specific characteristics of the two headsets used, what components they are made of, and why those types of headsets were chosen, as well as the price range. Moreover, for the sample that had no experience with VR headsets, it would be interesting to understand whether the environments they were subjected to were realistic or not. | **Line 274- 283:** The PICO 4 Enterprise headset retails at approximately £900 ([PICO 4 Enterprise EU (8G+256G) VR Headset (smartteck.co.uk)](about:blank). The second was an interactive video about marine wildlife called “Hydrous presents: EXPLORE” on HTC Vive ([The Hydrous presents: EXPLORE \| SideQuest (sidequestvr.com)](about:blank). This virtual environment had realistic images of the ocean. The HTC Vive headset costs £1,299 ([VIVE XR Elite - Convertible, All-in-One XR Headset \| VIVE United Kingdom](about:blank)). These headsets were previously owned by the research team. Both contain the standards features of VR such as, screen with a 360-degree view and hand controllers that allow the user to interact with the virtual environment. However, both the VR games are available on other cheaper VR headsets, such as Oculus Meta Quest 2, which costs £250 on average ([Meta Quest 2: Immersive all-in-one VR headset \| Meta Store \| Meta Store](about:blank)). |
